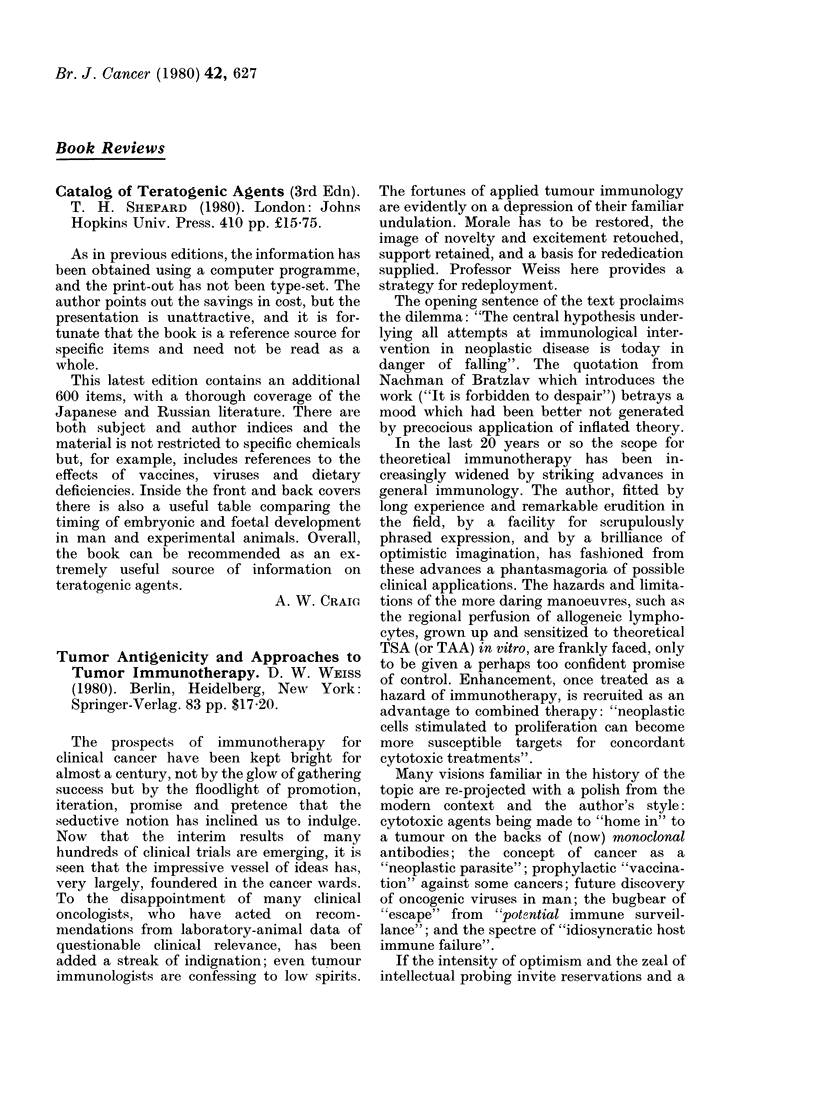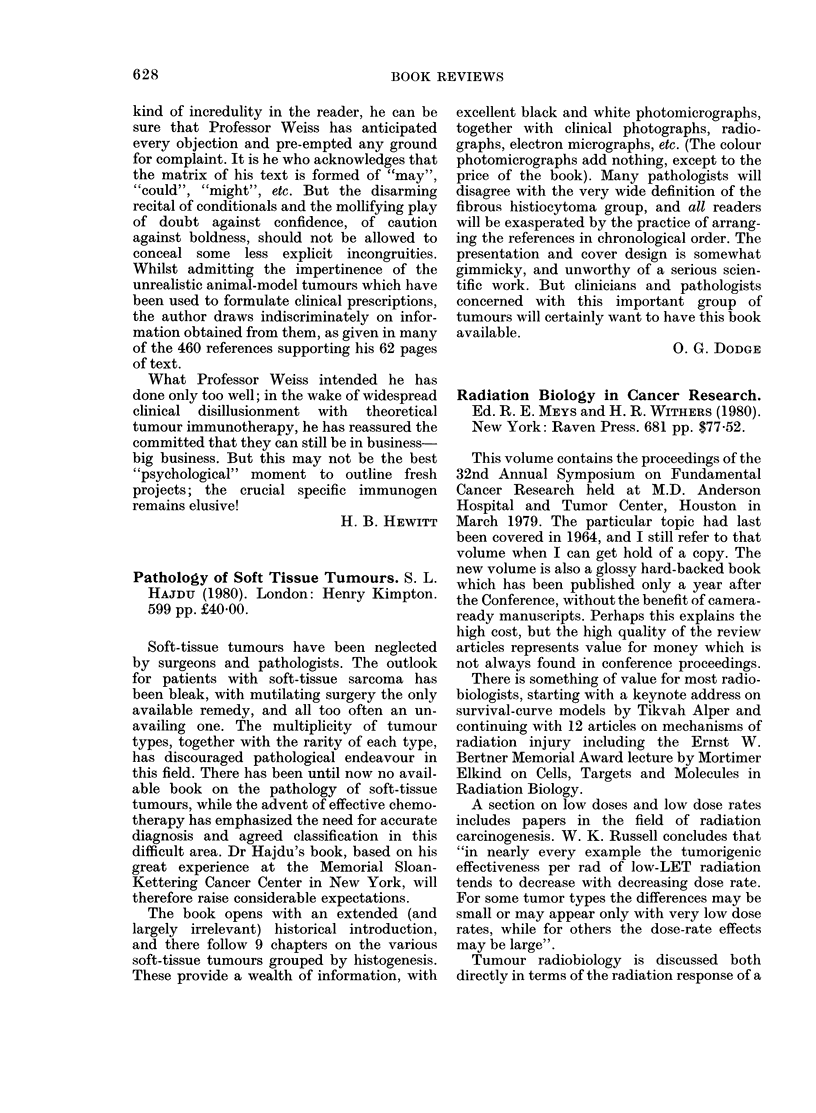# Tumor Antigenicity and Approaches to Tumor Immunotherapy

**Published:** 1980-10

**Authors:** H. B. Hewitt


**Tumor Antigenicity and Approaches to Tumor Immunotherapy.** D. W. WEISS (1980). Berlin, Heidelberg, New York: Springer-Verlag. 83 pp. $17·20.

The prospects of immunotherapy for clinical cancer have been kept bright for almost a century, not by the glow of gathering success but by the floodlight of promotion, iteration, promise and pretence that the seductive notion has inclined us to indulge. Now that the interim results of many hundreds of clinical trials are emerging, it is seen that the impressive vessel of ideas has, very largely, foundered in the cancer wards. To the disappointment of many clinical oncologists, who have acted on recommendations from laboratory-animal data of questionable clinical relevance, has been added a streak of indignation; even tumour immunologists are confessing to low spirits. The fortunes of applied tumour immunology are evidently on a depression of their familiar undulation. Morale has to be restored, the image of novelty and excitement retouched, support retained, and a basis for rededication supplied. Professor Weiss here provides a strategy for redeployment.

The opening sentence of the text proclaims the dilemma: "The central hypothesis underlying all attempts at immunological intervention in neoplastic disease is today in danger of falling". The quotation from Nachman of Bratzlav which introduces the work ("It is forbidden to despair") betrays a mood which had been better not generated by precocious application of inflated theory.

In the last 20 years or so the scope for theoretical immunotherapy has been increasingly widened by striking advances in general immunology. The author, fitted by long experience and remarkable erudition in the field, by a facility for scrupulously phrased expression, and by a brilliance of optimistic imagination, has fashioned from these advances a phantasmagoria of possible clinical applications. The hazards and limitations of the more daring manoeuvres, such as the regional perfusion of allogeneic lymphocytes, grown up and sensitized to theoretical TSA (or TAA) *in vitro*, are frankly faced, only to be given a perhaps too confident promise of control. Enhancement, once treated as a hazard of immunotherapy, is recruited as an advantage to combined therapy: "neoplastic cells stimulated to proliferation can become more susceptible targets for concordant cytotoxic treatments".

Many visions familiar in the history of the topic are re-projected with a polish from the modern context and the author's style: cytotoxic agents being made to "home in" to a tumour on the backs of (now) *monoclonal* antibodies; the concept of cancer as a "neoplastic parasite"; prophylactic "vaccination" against some cancers; future discovery of oncogenic viruses in man; the bugbear of "escape" from "*potential* immune surveillance"; and the spectre of "idiosyncratic host immune failure".

If the intensity of optimism and the zeal of intellectual probing invite reservations and a kind of incredulity in the reader, he can be sure that Professor Weiss has anticipated every objection and pre-empted any ground for complaint. It is he who acknowledges that the matrix of his text is formed of "may", "could", "might", *etc.* But the disarming recital of conditionals and the mollifying play of doubt against confidence, of caution against boldness, should not be allowed to conceal some less explicit incongruities. Whilst admitting the impertinence of the unrealistic animal-model tumours which have been used to formulate clinical prescriptions, the author draws indiscriminately on information obtained from them, as given in many of the 460 references supporting his 62 pages of text.

What Professor Weiss intended he has done only too well; in the wake of widespread clinical disillusionment with theoretical tumour immunotherapy, he has reassured the committed that they can still be in business—big business. But this may not be the best "psychological" moment to outline fresh projects; the crucial specific immunogen remains elusive!

H. B. HEWITT